# Transient versus stable nature of fear of falling over 24 months in community-older persons with falls– data of the EU SCOPE project on Kidney function

**DOI:** 10.1186/s12877-022-03357-0

**Published:** 2022-08-23

**Authors:** Ellen Freiberger, Paolo Fabbietti, Andrea Corsonello, Fabrizia Lattanzio, Rada Artzi-Medvedik, Robert Kob, Itshak Melzer, SCOPE consortium, Gerhard Wirnsberger, Regina Roller-Wirnsberger, Francesco Mattace-Raso, Lisanne Tap, Tomasz Kostka, Agnieszka Guligowska, Francesc Formiga, Rafael Moreno-González, Pedro Gil, Sara Laínez Martínez, Andreas Bekmann, Christian Weingart, Cornel Sieber, Johan Ärnlöv, Axel Carlsson, Sabine Britting

**Affiliations:** 1 Department of Internal Medicine-Geriatrics, Institute for Biomedicine of Aging (IBA), Friedrich-Alexander-Universität Erlangen-Nürnberg, Erlangen, Germany; 2 Italian National Research Center On Aging (IRCCS INRCA), Ancona, Fermo and Cosenza, Italy; 3 Department of Nursing, Recanati School for Community Health Professions at the Faculty of Health Sciences, Ben-Gurion University of the Negev, Beer-Sheva, Israel; 4 Maccabi Health Services, Israel; and Department of Nursing, Recanati School for Community Health Professions at the Faculty of Health Sciences, Ben-Gurion University of the Negev, Beer-Sheva, Israel; 5 Department of Physical Therapy, Recanati School for Community Health Professions at the Faculty of Health Sciences, Ben-Gurion University of the Negev, Beer-Sheva, Israel

**Keywords:** Fear of falling, Older people, Falls, Injurious falls, Physical function, Fallers, Osteoporosis

## Abstract

**Background:**

Fear of falling (FoF) is an important risk factor for falls among older people. The objectives of our investigations were: a.) to present characteristics of older community-dwelling (CD) fallers with persistent or transient FoF (P-FoF or T-FoF) over 12 months, and b.) to investigate clinical predictors of P-FoF and T-FoF and c.) to explore differences between P-FoF and T-FoF.

**Methods:**

Our series consisted of 389 older people reporting a fall or injurious fall at baseline and during 24 months follow-up participating in a multicenter prospective study. T-FoF was defined as participants reported “not at all” at baseline and “somewhat/fairly/very concerned” at follow-up, or “not at all” at follow-up, and “somewhat/fairly/very concerned” at baseline, and P-FoF was defined as participants answered “somewhat/fairly/very concerned” in both assessments at baseline and at follow-up. The association between risk factors and T-FoF or P-FoF was investigated by logistic regression analysis.

**Results:**

The mean age of fallers in our sample was 79.0 years (SD 6.0), and 54.2% were females. Out of 389 older adults with a fall history at baseline, 83 participants (21.3%) did not report any FoF over time, P-FoF and T-FoF were observed in 42.7% and 35.9% of participants, respectively. After adjusting for potential confounders (e.g. age, gender), osteoporosis (OR = 2.04, 95%CI = 1.03–4.05) and impaired physical performance (OR = 2.38, 95%CI = 1.12–5.03) were significant predictors of T-FoF vs No-FoF. Osteoporosis (OR = 2.68, 95%CI = 1.31–5.48), depressive symptoms (OR = 3.54, 95%CI = 1.23–10.1) and living alone (OR = 2.44, 95%CI = 1.17–5.06) were significantly associated with P-FoF vs No-FoF. When comparing T-FoF and P-FoF, female gender (OR = 1.95, 95%CI = 1.16–3.27), BMI (OR = 1.08, 95%CI = 1.02–1.14), overall comorbidity (OR = 1.07, 95%CI = 1.02–1.13) and depression (OR = 2.55, 95%CI = 1.33–4.88) were significant predictors of P-FoF.

**Conclusions:**

T-FoF and P-FoF may be predicted by different sets of risk factors among older fallers. Thus, fallers should be screened for FoF especially when carrying specific risk factors, including female gender, osteoporosis, depression, living alone, impaired physical performance, BMI, comorbidity. These findings may be helpful in designing tailored intervention to blunt the risks related to consequence of FoF among older people experiencing falls.

**Trial registration:**

The SCOPE study was registered prospectively at clinicaltrials.gov (NCT02691546; 25/02/2016).

**Supplementary Information:**

The online version contains supplementary material available at 10.1186/s12877-022-03357-0.

## Background

Due to the demographic changes, new challenges arise for the public health care systems and in older persons themselves. Falls and psychological concerns of falls are nowadays regarded as a threat to health, autonomy and mobility in older community-dwelling (CD) persons [1–4]. Past studies have determined that the risk factors of future falling can be intrinsic i.e., age, gender, reduced physical performance, and the presence of chronic diseases e.g. arthritis. Psychological conditions, such as fall-related psychological concerns (FrPC), also deserves to be mentioned. Among them, fear of falling (FoF) is especially relevant and may develop in older adults who have experienced an accidental fall [5] but is also prevalent in older persons without a falls history.

Fear of Falling (FoF) is part of the theoretical construct of FrPC which includes also the dimension of self-efficacy or balance related confidence [1]. FoF is defined as enduring concerns about future falls [6], and is common in older persons with and without history of falls, with prevalence rates ranging from 20–83% in CD older persons [7, 8]. Current evidence suggests that FoF is a multifactorial phenomenon, with female gender, older age and use of walking aids being involved as relevant risk factors [9]. On the other side, FoF is reported to be associated with activity restriction [10, 11], decline in physical function and mobility limitations [12–14]. In the study by Auis et al. [13] people with high and moderate FoF had about 3 times and 2.5 times higher risk of mobility disability, respectively, compared to those with no/low FoF. FoF is also related to depression [11] and cognitive impairments [15, 16]. Additionally, Chen et al. [17] recently reported that experiences of falling during the previous month or the previous year were significantly associated with a FoF. Therefore, a fall or injurious fall also threatens functionality and independence in older persons and is often the start of a downward spiral with nursing home admission or even death [18].

As FoF is more prevalent in fallers then in non-fallers [8] it is of importance to investigate the dynamics of FoF over time as it has been shown to be predictive of future falls [8]. Research demonstrated that FoF can be of transient or persistent nature [11, 19]. Persistent FoF (P-FoF) was related to worse physical performance and more reported falls [11]. Oh-Park et al. [19] raised the point that in geriatric medicine, dynamic transitions between different stages of functionality have gained more interest e.g. in frailty [20, 21] or mobility research [22]. Such a conceptual framework likely apply to FoF research, as short-term FoF might act as a preventive strategy [23] while P-FoF could result in the negative effects described above. From this point of view, investigating risk factors associated with transient FoF (T-FoF) or P-FoF would be useful in managing FoF and its consequences: identifying older persons at risk of developing P-FoF could help to interrupt the downhill spiral with all the negative effects down the road. Additionally, this will provide a clearer understanding of the dynamics of FoF over time in older people, and can deepen insight into the underlying FoF mechanisms and falls. In addition, it may be relevant to optimally address individuals’ needs and to design tailored preventive interventions.

As described above, only few studies have investigated the persistent or/and transient nature of FoF in older CD persons already having experienced a fall or injurious fall in a longitudinal study design [2, 11]. One might speculate that over time the detrimental experience of a fall could be reduced and that FoF could also decrease or even disappear. Data of the 2-year SCOPE study [24] provided us the opportunity to investigate the difference in older CD persons with a fall history and P-FoF or T-FoF. In the present study, we aimed at investigating characteristics of older CD fallers with P-FoF or T-FoF and No-FoF.

## Methods

### Study design and participants

Our sub-sample consisted of participants enrolled in the Screening of Chronic Kidney Disease (CKD) among CD Older People across Europe multicenter observational study (SCOPE). The SCOPE study (European Union Horizon 2020 program, Grant Agreement no. 436849), is a multicenter 2-year prospective cohort study investigating CKD involving patients older than 75 years attending outpatient services in participating institutions in Austria, Germany, Israel, Italy, the Netherlands, Poland and Spain. Methods of the SCOPE study have been extensively described elsewhere [24].

Overall, 2,461 subjects were initially enrolled in the study, but only 746 reported a fall or an injurious fall at baseline. Of them, 357 participants were excluded because they did not experience a fall at follow-up (FoF was only obtained in case of a new fall reported), thus leaving a final sample of 389 older adults with a fall (or injurious fall) at baseline and in at least one follow-up assessment (12 or 24 months).

### Ethics

Participants signed a written informed consent before entering the study. The study protocol was approved by ethics committees at all participating institutions, and complies with the Declaration of Helsinki and Good Clinical Practice Guidelines.

## Measures

Sociodemographic, anthropometry, laboratory analysis. Participant´s characteristics were assessed during the baseline interview and medical examination. Demographic variables included age, gender and self-reported educational level and marital status. Body mass index (BMI) was calculated by body weight and height and expressed as kg/m^2^. To assess the CKD stage, blood and urine analysis were performed by the locally certified laboratories adhering to the protocol. Follow-up data were obtained at 12 months and 24 months.

### Fear of Falling and Falls

FoF was obtained with a single question with possible answers ranging from “not at all concerned”, “somewhat concerned”, “fairly concerned” and “very concerned”.

FoF was categorized as follows:

- Absent (No-FoF): if participants answered “not at all” in both assessments at baseline and at follow-up;
- Transient (T-FoF): if participants reported “not at all” at baseline and “somewhat/fairly/very concerned” at follow-up, or “not at all” at follow-up, and “somewhat/fairly/very concerned” at baseline;
- Persistent (P-FoF): if participants answered “somewhat/fairly/very concerned” in both assessments at baseline and at follow-up.

Data on falls and incident falls were collected during baseline, 1-year and 2-year follow-up via a face-to face interview questionnaire. For prevalence of falls and injurious falls, two questions were asked: 1) how many times have you fallen in the past 12 months, (answer “No” for 0 events, and the answer “yes” for 1 or more falls), and 2) what kind of injuries did you sustain from a fall (answer: fractures, treated and untreated injury, and no injury). Further information of the location and circumstances of the fall was also documented. An injurious fall was defined as a fall causing fractures, treated or untreated injuries.

### Physical function

Physical function was assessed with the short physical performance battery (SPPB) [25] including gait speed, five chair-stands test and balance test. Each sub-test was conducted and then transformed to a score ranging between 0–4. The total score of the SPPB could sum up to 12 points, where higher scores represent better function. We included SPPB total score, and each score of the three domains separately to get information on the impact of lower-limbs strength, gait speed and balance on FoF.

### Comprehensive geriatric assessment

The Comprehensive Geriatric Assessment (CGA) was performed including Mini Mental State Examination (MMSE)/cognitive status [26], 15-items Geriatric Depression Scale (GDS)/mood [27], Basic (ADL) and Instrumental Activities of Daily Living (IADL)/self-reported disability [28, 29]. Overall comorbidity was assessed by using Cumulative Illness Rating Scale for Geriatrics (CIRS-G Total Score)/overall comorbidity [30]. Number of medications was also calculated and included in the analysis. Health related quality of life was rated by Euro-Qol 5D [31] visual scale asking participants to evaluate their overall health today on a vertical visual analogue scale, ranging from 0 “worst possible” to 100 “best possible”.

### Statistical analysis

First, descriptive analysis of the study population grouped according to FoF categories was provided. All variables were not normally distributed, therefore continuous data were expressed by median (interquartile range). Categorical data were reported as number (percentage). Chi-square test was used to analyze categorical variables, while Kruskal–Wallis non-parametric test was used for continuous ones. Bonferroni correction was applied when appropriate.

In order to study the correlates of T-FoF vs. No-FoF, P-FoF vs No-FoF, and P-FoF vs. T-FoF, logistic regression models adjusted for age and gender were built. Finally, fully adjusted models with age, gender and other significant variables were created. The explanation is that in age and gender adjusted models, you can see the OR of every variable, corrected for age and sex, while in fully adjusted models you can see the OR of every variable, corrected for age, sex and the other variables. Furthermore, in the fully adjusted model, adjusting also for ADL and MMSE did not change our results.

Statistical analysis was carried out using SPSS for Win V24.0 (SPSS Inc., Chicago, IL, USA). A *p*-value < 0.05 was considered statistically significant.

## Results

A total sample of 389 SCOPE participants were included in our analysis. The median age of our sample was 79.0 years (interquartile difference 6.0) and 247 were females (63.5%). Table 1 shows the characteristics of our participants. All participants had experienced at least one fall, and over 50% had experienced even an injurious fall. Compared to No-FoF group, people with T-FoF were more frequently users of walking aid and live alone. Additionally, T-FoF group also had greater prevalence of SPPB impairment, IADL dependency, osteoporosis, and depressive symptoms compared to No-FoF. The P-FoF group was characterized by greater prevalence of female gender, use of walking aid, living alone, SPPB impairment, IADL dependency, osteoporosis and depressive symptoms compared to No-FoF groups. Finally, people in the P-FoF group also showed the lowest Euro-QoL-5D visual scale and the highest BMI and CIRS values (Table 1).

**Table 1:** Demographic and characteristics of the study population (*N* = 389)

Variable	No-FoF (*n* = 83)	T- FoF (*n* = 140)	P- FoF (*n* = 166)	*p*-value
Age (years)	79.0 (7.0)	79.0 (6.0)	80.0 (6.0)	0.893
Gender (female), n (%)	45 (54.2)	83 (59.3)	119 (71.7)	**0.011 **^**bc**^
Any injurious fall, n (%)	46 (55.4)	85 (60.7)	115 (69.3)	0.075
Use of walking aid, n (%)	14 (16.9)	38 (27.1)	63 (38.0)	**0.002 **^**abc**^
Living alone, n (%)	17 (20.5)	41 (29.3)	59 (35.5)	**0.049 **^**abc**^
SPPB < 7, n (%)	13 (15.7)	48 (34.3)	66 (39.8)	**0.001 **^**ab**^
Quality of Life (EQoL-VAS) < = 75, n (%)	75.0 (25.0)	70.0 (29.0)	65.0 (25.0)	**0.001 **^**ac**^
BMI (kg/m^2^)	27.0 (6.0)	26.7 (5.3)	28.0 (5.7)	**0.006 **^**ac**^
Dependency in 1 or more IADL, n (%)	26 (31.3)	60 (42.9)	78 (47.0)	0.060
Dependency in 1 or more BADL, n (%)	2 (2.4)	13 (9.3)	16 (9.6)	0.108
MMSE < 24, n (%)	7 (8.4)	7 (5.0)	16 (9.6)	0.305
Cumulative Illness Rating Score (CIRS-G)	8.0 (7.5)	8.0 (6.0)	10.0 (7.0)	**0.004 **^**ac**^
Osteoporosis, n (%)	16 (19.3)	51 (36.4)	69 (41.6)	**0.002 **^**ab**^
GDS score > 5, n (%)	5 (6.0)	16 (11.4)	46 (27.7)	** < 0.001 **^**abc**^

***Abbreviations*:** *No-FoF* no Fear of Falling, *T-FoF* Transient Fear of Falling, *P-FoF* Persistent Fear of Falling, *BMI* body mass index, *GDS* geriatric depression scale, *IADL* instrumental activities of daily living, *SSPB* short physical performance battery, *EQoL-VAS* visualisation scale of EuroQoL-5D, *MMSE* Minimal State Examination

^a^ *p* < 0.05 for No-FoF versus T- FoF

^b^ *p* < 0.05 for No-FoF versus P- FoF

^c^ *p* < 0.05 for T- FoF versus P- FoF

### Multivariate logistic regression

After adjusting for potential confounders, only SPPB impairment (OR = 2.38, 95%CI = 1.12–5.03) and osteoporosis (OR = 2.04, 95%CI = 1.03–4.05) were significantly associated with T-FoF compared to No-FoF (Table 2).

**Table 2:** Correlates of T-FoF vs. No-FoF among older adults aged 75 and older (*n* = 223)

Predictors	OR (95%CI) T-FoF Crude Model	OR (95%CI) T-FoF Age and gender adjusted Model ^*^	OR (95%CI) T-FoF Fully adjusted Model ^*^
Age [years]	0.99 (0.93 – 1.05)	0.99 (0.93 – 1.06)	0.97 (0.90 – 1.04)
Gender (female)	1.23 (0.71 – 2–13)	1.22 (0.70 – 2.12)	1.22 (0.64 – 2.31)
Dependency in 1 or more IADL, n (%)	1.64 (0.93 – 2.91)	**1.99 (1.06 – 3.74)**	1.48 (0.76 – 2.91)
SPPB < 7	**2.81 (1.41 – 5.86)**	**3.07 (1.51 – 6.23)**	**2.38 (1.12 – 5.03)**
Osteoporosis	**2.40 (1.26 – 4.57)**	**2.38 (1.23 – 4.63)**	**2.04 (1.03 – 4.05)**

***Abbreviations*:** *No-FoF* no Fear of Falling, *T-FoF* Transient Fear of Falling, *IADL* instrumental activities of daily living, *SSPB* short physical performance battery, *EQoL-VAS* visualisation scale of EuroQoL-5D

^*^ in age and gender adjusted models, the OR of every variable, corrected for age and sex is calculated, while in fully adjusted models the OR of every variable, corrected for age, sex and the other variables is calculated

At variance, osteoporosis (OR = 2.68, 95%CI = 1.31–5.48), depressive symptoms (OR = 3.54, 95%CI = 1.23–10.1) and living alone (OR = 2.44, 95%CI = 1.17–5.06) were significantly associated with P-FoF (Table 3).

**Table 3:** Correlates of P-FoF (vs No-FoF) among older adults aged 75 and older (*n* = 249)

Predictors	OR (95%CI) P-FoF Crude Model	OR (95%CI) P-FoF Age and gender adjusted Model ^*^	OR (95%CI) P-FoF Fully adjusted Model ^*^
Age [years]	1.00 (0.95 – 1.06)	1.02 (0.96 – 1.08)	0.98 (0.91 – 1.05)
Gender (female)	**2.14 (1.24 – 3.70)**	**2.20 (1.26 – 3.85)**	1.63 (0.79 – 3.40)
BMI [kg/m^2^]	**1.07 (1.01 – 1.14)**	**1.08 (1.01 – 1.14)**	1.06 (0.99 – 1.14)
Dependency in 1 or more IADL, n (%)	**1.94 (1.12 – 3.38)**	**2.52 (1.37 – 4.64)**	1.69 (0.82 – 3.46)
Quality of Life (EQoL-VAS) < = 75	**2.53 (1.45 – 4.44)**	**2.51 (1.42 – 4.43)**	1.86 (0.97 – 3.59)
SPPB < 7	**3.55 (1.82 – 6.93)**	**3.44 (1.73 – 6.81)**	1.45 (0.62 – 3.38)
Osteoporosis	**2.98 (1.59 – 5.57)**	**2.59 (1.36 – 4.93)**	**2.68 (1.31 – 5.48)**
CIRS-G	**1.07 (1.01 – 1.14)**	**1.09 (1.03 – 1.16)**	1.03 (0.96 – 1.10)
GDS > 5	**5.98 (2.28 – 15.7)**	**5.67 (2.14 – 15.0)**	**3.54 (1.23 – 10.1)**
Use of walking aid	**3.01 (1.57 – 5.80)**	**3.17 (1.58 – 6.37)**	1.76 (0.76 – 4.04)
Injurious falls	**1.81 (1.05 – 3.12)**	**1.78 (1.02 – 3.09)**	1.62 (0.85 – 3.09)
Living alone	**2.14 (1.15 – 3.98)**	**1.89 (1.00 – 3.56)**	**2.44 (1.17 – 5.06)**

***Abbreviations*:** *No-FoF* no Fear of Falling, *P-FoF* persistent Fear of Falling, *BMI* body mass index, *GDS* geriatric depression scale, *IADL* instrumental activities of daily living, *SSPB* short physical performance battery, *EQoL-VAS* visualisation scale of EuroQoL-5D, *CIRS* Cumulative Illness Rating Score

^*^ in age and gender adjusted models, the OR of every variable, corrected for age and sex is calculated, while in fully adjusted models the OR of every variable, corrected for age, sex and the other variables is calculated

Finally, in the comparison between the T-FoF and P-FoF group, the fully adjusted model showed that female gender (OR = 1.95, 95%CI = 1.16–3.27), BMI (OR = 1.08, 95%CI = 1.02–1.14), overall comorbidity (OR = 1.07, 95%CI = 1.02–1.13) and depressive symptoms (OR = 2.55, 95%CI = 1.33–4.88) were significantly associated with P-FoF (Table 4).

**Table 4:** Correlates of P-FoF (vs T-FoF) among older adults aged 75 and older (*n* = 306)

Predictors	Crude Model OR (95%CI)	Age and gender adjusted Model ^*^ OR (95%CI)	Fully adjusted Model ^*^ OR (95%CI)
Age [years]	1.01 (0.96 – 1.07)	1.02 (0.97 – 1.08)	1.01 (0.96 – 1.07)
Gender (female)	**1.74 (1.08 – 2.80)**	**1.77 (1.10 – 2.88)**	**1.95 (1.16 – 3.27)**
BMI [kg/m^2^]	**1.09 (1.03 – 1.15)**	**1.09 (1.04 – 1.15)**	**1.08 (1.02 – 1.14)**
CIRS-G	**1.08 (1.03 – 1.13)**	**1.09 (1.04 – 1.15)**	**1.07 (1.02 – 1.13)**
GDS > 5	**2.97 (1.59 – 5.53)**	**2.77 (1.48 – 5.20)**	**2.55 (1.33 – 4.88)**

***Abbreviations*:** *P-FoF* persistent Fear of Falling, *T-FoF* Transient Fear of Falling, *BMI* body mass index, *GDS* geriatric depression scale, *CIRS* Cumulative Illness Rating Score

^*^ in age and gender adjusted models, the OR of every variable, corrected for age and sex is calculated, while in fully adjusted models the OR of every variable, corrected for age, sex and the other variables is calculated

## Discussion

The present study shows that T-FoF and P-FoF may be characterized by different risk factor profiles in a population of older CD people who experienced at least one fall at the baseline assessment. Indeed, while only osteoporosis and impaired physical performance may be associated with T-FoF vs No-FoF, depressive symptoms, living alone and osteoporosis may characterize people with P-FoF vs No-FoF. Finally, female gender, BMI, overall comorbidity and depressive symptoms were significantly associated with P-FoF when compared with T-FoF.

Overall, our findings confirm the dynamic complexity of FoF among older people and strengthen the need to obtain longitudinal data about this clinically relevant topic potentially exposing older people to a downhill spiral of functional decline [4, 5]. Indeed, we could compare our results with few available longitudinal studies only to some extent.

Oh-Park et al. defined T-FoF as new onset FoF, and P-FoF as reporting FoF at two or more interviews over a 24-months’ time period in a general population of older people [19], while only older fallers were included in our study. Methodological differences are likely to explain the somewhat different incidence of P-FoF observed in the two studies (60% vs 42.7%) [19].

Another longitudinal study, Hispanic Established Populations for the Epidemiologic Study of the Elderly (H-EPESE) investigated FoF levels with a single question comparable to our method in a general older population including both fallers and non-fallers over a 10-years’ time period [32]. They grouped older adults into two groups (no FoF and FoF). The FoF group was further differentiated between moderate and severe level of FoF [32]. Severe FoF was found to increase by 15.6% from the baseline assessment to the last wave after 10 years [32], and 31.2% reported no FoF at the end of the 10 years follow-up, which is higher than in our study, mainly because we selected a population of fallers, and suggests that FoF may be strongly influenced by recent event of falling and that FoF may decrease over time. Finally, another longitudinal study investigated the change of FoF levels over a period of 3 years in CD older women [11]. Although the methods of obtaining FoF differed (i.e., three questions related to FoF), 33% reported FoF at baseline. Over the follow up period of three years, 21% did not report any symptoms of FoF but the percentage of participants reporting any symptoms of FoF increased to 46% [11]. Although Austin et al. did not label the groups with regard to persistent, transient or No-FoF they could also demonstrate the transient nature of FoF levels by reporting a group decreasing and increasing FoF level over time.

The different profiles of risk factors characterizing T-FoF and P-FoF also deserve to be discussed. Overall, our study support already reported risk factors for developing FoF over time in the literature [7, 9, 33], with physical limitations and sex in the front but also living alone playing a role. Osteoporosis was a significant predictor of both T-FoF and P-FoF compared to No-FoF, which is in keeping with the notion that osteoporosis is a relevant risk factor for FoF [34–36]. Additionally, the awareness of risk related to falls and fractures may be a major trigger of FoF among older people, which in turn may lead to reduced physical activity and consequent downhill spiral of functional decline [35].

The variable “living alone” is related to P-FoF. The situation of living alone has been investigated in relationship to FoF in general [9]. The situation of living alone may support the P- FoF level. The older person is aware of being at higher risk of falling as in hazardous situation no one else can come to help. As this situation remains consistent the P-FoF level will not change without effective intervention but create probably the above mentioned negative downhill spiral.

Depression was also related to FoF in other studies [2, 9, 37] although not all studies had included fallers and investigated P-FoF in CD older persons. Older persons with depression often avoid activities or social interaction, which in turn might influence the functional status by decreasing strength or balance and increase risk of falling [38]. As depression characterized P-FoF as well as T-FoF, it appears that depressive symptoms may be one key factor to experience FoF. Therefore, as depression is part of the routinely obtained geriatric assessment FoF should also be routinely screened.

Our findings of differences between T-FoF and P-FoF is supported by a recent analysis, classification and regression tree (CaRT) [33]. This approach revealed 12 different end groups with a minimum of two and a maximum of five predictors [33] demonstrating the need for a more differentiated approach in the nature of FoF over time, and showing the complexity in the identification process of older persons with FoF. The recognition of components contributing to the dynamic nature of FoF is crucial for effective interventions.

Our findings have several clinical implications. Firstly, different predictors underline the importance of routinely screening of FoF in the primary care setting in older CD persons to identify the “high-risk” population for P-FoF and prevent the downhill spiral with further mobility or physical function decline. Secondly, much more research is needed with regard of investigating the group of T- FoF, as the level of FoF can either increase or even decrease over time. Unfortunately, our data did not allow us to investigate this aspect as well. One explanation for a decreasing level of FoF could be that the experience of falls is fading in the memory, and the older person is gaining more self-confidence in their balance again. A further explanation could be that the older person is paying less attention to hazardous situation and thus decreasing his/her level of FoF. An increasing level of FoF could actually fuel the downhill spiral by decreasing daily functional and mobility activities thus increasing physical limitations. Thirdly, our study supports the need for multicomponent interventions adopted probably to the right type of FoF for being effective. Evidence on effective interventions are heterogeneous. A systematic review has demonstrated no positive effects of exercise based FoF interventions until now [39] whereas a recent systematic review has demonstrated positive effects [40]. Therefore, the best type of interventions and the optimal individual components are still unknown.

### Limitations and strengths

The main limitation of our study is that we could only include older CD participants reporting a fall over the two years’ time period. Therefore, our results might not be generalizable to all older CD persons. In addition, we did not differentiate between falls and injurious falls in our statistical analyses which might have had an impact on the risk factors for the T-FoF and P-FoF groups. Another limitation, one might say that the method of obtaining FoF level using just a simple question with a Likert-Scale is problematic. Nevertheless, this methods had shown its reliability and usefulness in epidemiological studies [36]. Furthermore, one could argue that the single item question is clinically easy to administer and might be more robust than using tools that assess falls-efficacy which are commonly used in the field, yet they are distinct concepts.

A strength of our study is the description and investigation of the transient nature of FoF in older CD fallers, which is less investigated but needs more attention in future research and the clinical setting. In addition, our study includes a relatively large data of a European study in 8 different countries. Another strength of our study is the large set of potential cofounders including comprehensive geriatric assessment, and a 24-month follow-up longitudinal design in the context of small number of longitudinal data already published on such a relevant topic.

## Conclusions

Our study underlines the importance to investigate FoF not only cross-sectional but longitudinal to identify the high risk group of older CD persons with P-FoF. They are at risk of reducing their daily activities and thus fueling a downhill spiral on physical function, social isolation, reduced quality of life and even death. We also could identify that physical function is playing an important role in the identification of the T-FoF group. Furthermore, older CD persons with osteoporosis should routinely be screened for FoF as this was prevalent in both FoF groups.

In the present study we investigated the dynamics of FoF over a 12-months period in older CD persons with a falls history at baseline, by comparing the No-FoF's, T-FoF´s and P-FoF´s. This article follows our cross-sectional investigation about risk factors of FoF in older participants enrolled in the SCOPE trial [41].

Only 83 participants with a fall history at baseline (21.33%) did not report any FoF over time. In contrast, 166 participants (42.67%) reported P-FoF over time, and 140 participants (35.9%) reported T-FoF. Our longitudinal reported prevalence of FoF – ranging between the two groups from 35.9% to 42.67% – are consistent with the findings by a recent systematic review [36], demonstrating that for FoF levels obtained with a single question, the prevalence is somewhat lower compared to questionnaires. 

## Supplementary Information

**Additional file 1. **

## Data Availability

Data will be available for SCOPE consortium on request from the principal investigator, Fabrizia Lattanzio, Italian National Research Center on Aging (IRCCS INRCA), Ancona, Fermo and Cosenza, Italy. F.Lattanzio@inrca.it.

## Abbreviations

CI: Confidence Interval
CKD: Chronic Kidney Disease
FoF: Fear of Falling
FrPC: Fall-related psychological concern
GDS: Geriatric Depression Scale
IADL: Instrumental Activity of Daily Living
MMSE: Mini-Mental State Examination
OR: Odds ratio
SPPB: Short Physical Performance Battery
