# Supplementary material for: Transient versus stable nature of fear of falling over 24 months in community-older persons with falls– data of the EU SCOPE project on Kidney function
Source: BMC Geriatr. 2022 Aug 23;22:698. doi: 10.1186/s12877-022-03357-0 (PMC9396801; doi:10.1186/s12877-022-03357-0)
Supplement: Supplementary file 1 — Additional file 1. [file 12877_2022_3357_MOESM1_ESM.docx]

Supplementary Information: Full list of members of SCOPE consortium and their affiliations.

Fabrizia Lattanzio^2^, Paolo Fabbietti^2^, Andrea Corsonello^2^, Gerhard Wirnsberger^6^, Regina Roller-Wirnsberger^6^, Francesco Mattace-Raso^7^, Lisanne Tap^7^, Tomasz Kostka^8^, Agnieszka Guligowska^8^, Rada Artzi-Medvedik^3,4^, Itshak Melzer^5^, Francesc Formiga^9^, Rafael Moreno-González^9^, Pedro Gil^10^, Sara Laínez Martínez^10^, Ellen Freiberger^1^, Robert Kob^1^, Sabine Britting^1^, Andreas Bekmann^11^, Christian Weingart^11^, Cornel Sieber^1,12^, Johan Ärnlöv^13^, Axel Carlsson^13^, Silvia Bustacchini^2^, Silvia Bolognini^2^, Paola D’Ascoli^2^, Raffaella Moresi^2^, Giuseppina Di Stefano^2^, Cinzia Giammarchi^2^, Anna Rita Bonfigli^2^, Roberta Galeazzi^2^, Federica Lenci^2^, Stefano Della Bella^2^, Enrico Bordoni^2^, Mauro Provinciali^2^, Robertina Giacconi^2^, Cinzia Giuli^2^, Demetrio Postacchini^2^, Sabrina Garasto^2^, Annalisa Cozza^2^, Francesco Guarasci^2^, Sonia D’Alia^2^, Romano Firmani^2^, Moreno Nacciariti^2^, Mirko Di Rosa^2^, Carolin Herzog^6^, Sonja Lindner^6^, Gijsbertus Ziere^7^, Jeannette Goudzwaard^7^, Łukasz Kroc^8^, Bartłomiej K Sołtysik^8^, Małgorzata Pigłowska^8^, Agnieszka Wójcik^8^, Zuzanna Chrząstek^8^, Natalia Sosowska^8^, Anna Telążka^8^, Joanna Kostka^8^, Elizaveta Fife^8^, Katarzyna Smyj^8^, Kinga Zel^8^, Yehudit Melzer^4^, Mark Clarfield^4^, Ilan Yehoshua^4^, Xavier Corbella^9^, Yurema Martínez^9^, Carolina Polo^9^, Josep Maria Cruzado^9^, Mónica González Alonso^10^, Jose A. Herrero Calvo^10^, Fernando Tornero Molina^10^, Lara Guardado Fuentes^10^, Pamela Carrillo García^10^, María Mombiedro Pérez^10^, Alexandra Renz^11^, Susanne Muck^1^, Stephan Theobaldy^11^, Revekka Kaltsa^1^, Tobias Feldreich^13^.

^1^Department of Internal Medicine-Geriatrics, Institute for Biomedicine of Aging (IBA), Friedrich-Alexander-Universität Erlangen-Nürnberg, Erlangen, Germany.

^2^Italian National Research Center on Aging (IRCCS INRCA), Ancona, Fermo and Cosenza, Italy.

^3^Department of Nursing, Recanati School for Community Health Professions at the faculty of Health Sciences, Ben-Gurion University of the Negev, Beer-Sheva, Israel.

^4^Maccabi Health Services, Israel; and Department of Nursing, Recanati School for Community Health Professions at the faculty of Health Sciences, Ben-Gurion University of the Negev, Beer-Sheva, Israel.

^5^Department of Physical Therapy, Recanati School for Community Health Professions at the faculty of Health Sciences, Ben-Gurion University of the Negev, Beer-Sheva, Israel.

^6^Department of Internal Medicine, Medical University of Graz, Austria.

^7^Section of Geriatric Medicine, Department of Internal Medicine, Erasmus MC, University Medical Center Rotterdam, The Netherlands.

^8^Department of Geriatrics, Healthy Ageing Research Centre, Medical University of Lodz, Poland.

^9^Geriatric Unit, Internal Medicine Department and Nephrology Department, Hospital Universitari de Bellvitge, Institut d’Investigació Biomèdica de Bellvitge - IDIBELL, L'Hospitalet de Llobregat, Barcelona, Spain.

^10^Department of Geriatric Medicine, Hospital Clínico San Carlos, Madrid, Spain.

^11^Department of General Internal Medicine and Geriatrics, Institute for Biomedicine of Aging, Krankenhaus Barmherzige Brüder, Friedrich-Alexander-Universität Erlangen-Nürnberg, Regensburg, Germany.

^12^Department of Medicine, Kantonsspital Winterthur, Winterthur, Switzerland.

^13^Department of Medical Sciences, Uppsala University, Sweden.

***Scientific advisory board (SAB)***

Roberto Bernabei, Catholic University of Sacred Heart, Rome, Italy

Christophe Bula, University of Lausanne, Switzerland

Hermann Haller, Hannover Medical School, Hannover, Germany

Carmine Zoccali, CNR-IBIM Clinical Epidemiology and Pathophysiology of Renal Diseases and Hypertension, Reggio Calabria, Italy

***Data and Ethics Management Board (DEMB)***

Dr. Kitty Jager, University of Amsterdam, The Netherlands

Dr. Wim Van Biesen, University Hospital of Ghent, Belgium

Paul E. Stevens, East Kent Hospitals University NHS Foundation Trust, Canterbury, United Kingdom
